# Semi‐quantitative duplex RT‐PCR reveals the low occurrence of Porcine Pegivirus and Atypical Porcine Pestivirus in diagnostic samples from the United States

**DOI:** 10.1111/tbed.13154

**Published:** 2019-03-21

**Authors:** Fangzhou Chen, Todd P. Knutson, Eli Braun, Yin Jiang, Stephanie Rossow, Douglas G. Marthaler

**Affiliations:** ^1^ Department of Veterinary Population Medicine College of Veterinary Medicine University of Minnesota Saint Paul Minnesota; ^2^ State Key Laboratory of Agricultural Microbiology College of Veterinary Medicine Huazhong Agricultural University Wuhan Hubei China; ^3^ Veterinary Diagnostic Laboratory College of Veterinary Medicine Kansas State University Manhattan Kansas

**Keywords:** atypical porcine pestivirus, Pegivirus, phylogenetics

## Abstract

Porcine Pegivirus (PPgV) and Atypical Porcine Pestivirus (APPV) are two recently identified porcine viruses. In this study, the identification of two viruses by metagenomic sequencing, and a duplex semi‐quantitative RT‐PCR was developed to detect these pathogens simultaneously. The PPgV strain Minnesota‐1/2016 had a 95.5%–96.3% nucleotide identity and clustered with the recently identified US PPgV strains, which is a distant clade from the German PPgV strains. The APPV strain Minnesota‐1/2016 shared an 87.3%–92.0% nucleotide identity with the other global APPV strains identity but only shared an 82.8%–83.0% nucleotide identity with clade II consisting of strain identified in China. Detection of both PPgV and APPV was 9.0% of the diagnostic cases. Co‐infection of PPgV and APPV was identified in 7.5% of the diagnostic cases. The occurrence and genetic characterization of PPgV and APPV further enhance our knowledge regarding these new pathogens in the United States.

## 1 INTRODUCTION

The *Pegivirus* genus, together with *Flavivirus*,* Hepacivirus* and *Pestivirus* genera, are members of the family Flaviviridae (Lindenbach, Murray, Thiel, & Rice, 2013). Pegiviruses are divided into 11 species (*Pegivirus A‐K*) (Smith et al., 2016) and have been identified from a variety of mammalian species including human, baboons, chimpanzees, monkeys, tamarins, bats, camels, horses, rodents and pigs (Tang et al., 2018; Thézé, Lowes, Parker, & Pybus, 2015). Pegiviruses have been detected in North and South America, Africa, Asia and Europe, indicating global presence of this virus (Bailey et al., 2016; de Souza et al., 2015; N'Guessan et al., 2018; Thézé et al., 2015; Van Nguyen et al., 2018). In 2016, Porcine Pegiviruses (PPgV) were discovered in pigs from Germany using Next Generation Sequencing (NGS) of serum from asymptomatic animals, and subsequent PCR testing detected PPgV in 2.2% of serum samples (Baechlein et al., 2016). Recently, PPgV was identified in the US, and 15.1% of the screened samples were positive for PPgV (Yang et al., 2018).

In 2015, a novel genetically distinct pestivirus, designated as Atypical Porcine Pestivirus (APPV), was identified via NGS in US pigs with congenital tremor (CT) (Hause et al., 2015). The following year, an experiential inoculation of APPV demonstrated CT in piglets (Arruda et al., 2016). Subsequently, APPV has been identified in porcine CT cases from Austria, Brazil, Canada, China, Germany, Hungary, the Netherlands, Spain and Sweden (Arruda et al., 2016; de Groof et al., 2016; Dénes et al., 2018; Dessureault, Choinière, Provost, & Gagnon, 2018; Gatto et al., 2018; Muñoz‐González et al., 2017; Pan et al., 2018; Postel et al., 2016; Shen et al., 2018; Yuan et al., 2017; Zhang et al., 2017, 2018). In China, two distant groups of APPV have been identified based on the coding sequences (CDS) of APPV strains (Shen et al., 2018).

Our study identified PPgV and APPV by NGS, leading to the development a semi‐quantitative RT‐PCR (sqRT‐PCR) for these viruses to determine the occurrence of PPgV and APPV in diagnostic samples from the US. The PPgV and APPV strains from the NGS samples were phylogenetically compared to the globally available strains.

## 2 MATERIALS AND METHODS

During August–September of 2016, two cases were submitted to the University of Minnesota Veterinary Diagnostic Laboratory (UMVDL) to determine the causative agent of disease. Case 1 consisted of 10 serum samples from a sow farm in Minnesota experiencing an increasing incidence of mummified and stillborn foetuses. The case was negative for routinely tested porcine pathogens. Case 2 consisted of oral fluid and was selected for NGS to discover new pathogens. The samples from these two cases were processed and submitted for library preparation and NGS on the Illumina MiSeq platform, using previously described methods (Jarvis et al., 2016). The raw data were analysed using in‐house NGS pipeline with custom bioinformatics tools (Knutson, Velayudhan, & Marthaler, 2017). Nucleotide and amino acid alignments were constructed using MAFFT in Geneious v9.1.8, and phylogenetic trees were built using PMYML with a GTR substitution model and bootstrapped with 500 replicates.

A duplex semi‐quantitative RT‐PCR (sqRT‐PCR) was developed for simultaneous detection of PPgV and APPV. The PPgV primers and probe were designed based on the available PPgV strains (KU351669–KU351671) at the time of the study and the PPgV sequence from our laboratory (KY798013). The APPV primers and probe were designed based on the 11 APPV strains available at the time of this study (KY624591, KX929062, KX778724, KU041639, KY612413, KX950762, KX950761, KY652092, LT594521, KU194229, KR011347) and the APPV sequence from our laboratory (MF590069). The PPgV and APPV primer and probe sets (Table S1) utilized the TaqMan Fast Virus 1‐Step Master Mix using recommended guidelines (Thermo Fisher, Austin, TX) and 8 μl of RNA. PCR was run in the 7500 Fast Real‐Time PCR System (Thermo Fisher) with the following thermal cycling conditions: reverse transcription (RT), 50°C for 5 min; RT inactivation/initial denaturation, 95°C for 20 s; followed by 40 cycles of denaturation at 95°C for 3 s and annealing at 60°C for 30 s. The analytical sensitivity was determined using custom gBlock (IDT, Skokie, IL) containing the PPgV and APPV targets while the analytical specificity was determined with 58 swine pathogens (Figure S1). The duplex sqRT‐PCR was used to test additional clinical diagnostic cases of serum or tissue from US swine herds, which were submitted to the UMVDL for routine diagnostics or surveillance for common porcine pathogens.

## 3 RESULTS

**Figure 1:**
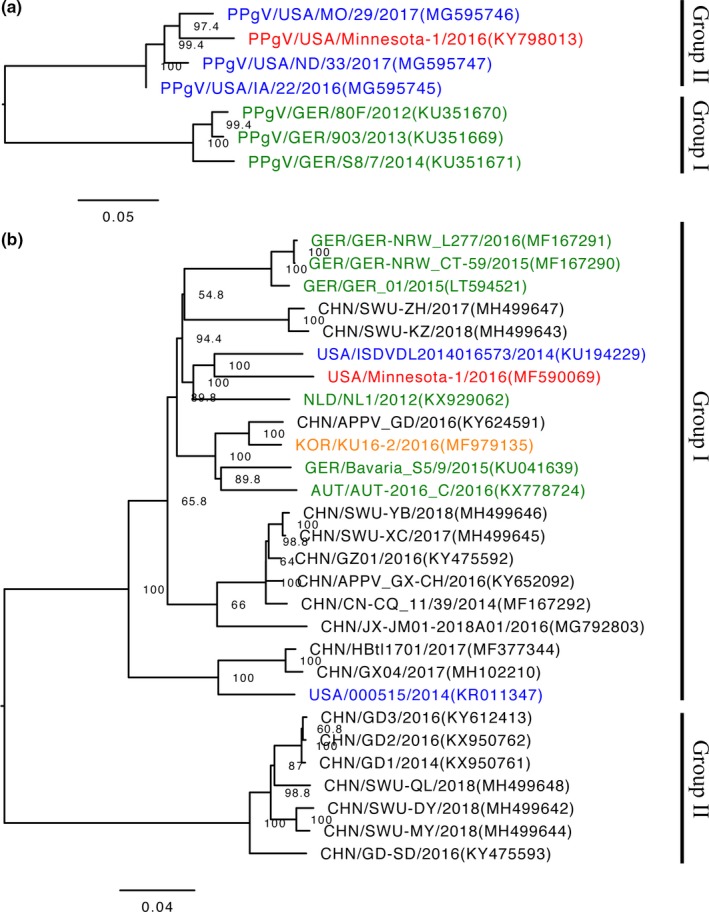
Phylogenetic tree of the complete coding sequences of Porcine Pegivirus (PPgV) (a) and Atypical Porcine Pestivirus (APPV) (b). GenBank accession numbers are in parentheses. The scale bars indicate nucleotide substitutions per site. The PPgV and APPV strains identified in this study are coloured red, strains from the US, Europe, Korea and China are coloured blue, green, orange and black respectively [Colour figure can be viewed at http://www.wileyonlinelibrary.com/]

In case 1, 10 serum samples were negative for routinely tested porcine pathogens by PCR and viral isolation, and PPgV was the only virus detected in two of the 10 samples (samples 4 and 6), using NGS. Only 173 pegivirus reads were identified in sample 4, whereas 509 reads were identified as pegivirus in sample 6. Using de novo assembly methods of the PPgV reads from sample 6, two PPgV contigs were obtained and was closed using Sanger sequencing, generating the strain PPgV/Pig‐wt/USA/Minnesota‐1/2016. The genome sequence length of PPgV Minnesota‐1/2016 was 9,727 nucleotides and had two deletions in the 5′UTR of compared with PPgV_903/Ger/2013 (deletion of an A at position 390 and TTC at position 591–593). Insertions or deletions were lacking in the CDS and 3′UTR. The Minnesota‐1 PPgV strain had a 95.5%–96.3% nucleotide and 98.8%–99.1% amino acid identities of the CDS to the US PPgV strains and had lower nucleotide and amino acid identities to the German strains (87.6%–88.9% and 96.2%–96.4% respectively) (Table 1). In phylogenetic tree, Minnesota‐1 strain clustered with other US strains (Group II), which was divergent from the previously reported German PPgV strains (Group 1) (Figure 1a).

**Table 1:** Nucleotide and amino acid identities of Minnesota strains to Groups I and II

	Group I		Group II	
	Nucleotide	Amino acid	Nucleotide	Amino acid
PPgV Minnesota‐1	95.5%–96.3%	98.8%–99.1%	87.6%–88.9%	96.2%–96.4%
APPV Minnesota‐1	87.3%–92.0%	94.0%–96.3%	82.8%–83.0%	91.4%–92.0%

In case 2, APPV was the only pathogenic virus detected. The APPV‐specific reads were de novo assembled, and a single contig representing the APPV genome of 11,397 nucleotides was generated (APPV/Pig‐wt/USA/Minnesota‐1/2016). The APPV strain Minnesota‐1/2016 had 87.3%–92.0% nucleotide and 94.0%–96.3% amino acid identities to global APPV genomes in GenBank, but an 82.8–83.0 nucleotide and 91.4%–92.0% amino acid identity to a recently identified clade of APPV strains from China (Table 1). In the phylogenetic tree, Minnesota‐1/2016 clustered in the same branch with the other APPV strains from Asia, Europe and the US (Group I). Group II remained unchanged composed only of strains from China (Figure 1b).

The identification of these two viruses in our NGS samples led to the development of duplex sqRT‐PCR, which had an analytic sensitivity of 7.5 and 75.2 copies per reaction for APPV and PPgV, respectively, and a 100% analytical specificity. Of the 67 clinical cases, 17 (25.0%) were positive for PPgV, APPV or co‐infected with PPgV and APPV (Table 2). PPgV was detected in six cases (9.0%) located from Minnesota, Iowa and Oklahoma. APPV was detected in six cases (9.0%), located from Minnesota, Iowa, South Dakota and Kentucky. Co‐infections of PPgV and APPV were identified in five cases (7.5%), located from Minnesota, South Dakota and Kentucky. Interestingly, only serum cases were positive for co‐infections of PPgV and APPV. In addition, eight of the 12 serum cases (66.7%) were positive for PPgV, whereas only seven of the 66 tissue cases (10.6%) were positive for PPgV. For APPV, seven of the 12 serum cases (58.3%) were positive, whereas only four of the 11 tissue cases (7.2%) were positive for APPV.

**Table 2:** sqRT‐PCR results for PPgV and APPV organized by specimen and state

State	PPgV	APPV	PPgV/APPV	Negative	Total
*Serum*					
Kentucky		1	1		2
Minnesota	2		2	2	6
Oklahoma	1				1
South Dakota		1	2		3
Total	3	2	5	2	12
*Tissues*					
Iowa	1	1		8	10
Ohio				3	3
Oklahoma				1	1
Minnesota	2	3		30	35
Missouri				6	6
Total	3	4	0	48	55
Total	6	6	5	50	67

## 4 DISCUSSION

The pathogenicity and role of pegiviruses as causative agents of diseases are host species dependent. Human pegivirus is prevalent in 5% of the world's population and was reported as co‐infections with other viruses, such as hepatitis C virus and HIV infected individuals (Berg et al., 2015; Jordier et al., 2018; N'Guessan et al., 2018; Schwarze‐Zander, Blackard, & Rockstroh, 2012; Wang et al., 2018). In addition, human pegivirus strains can be associated with febrile illness, haemophilia, acute mycocarditis and lymphoma (Bijvand et al., 2018; Fama et al., 2018; Takeuchi et al., 2018; Williams et al., 2018). In horses, a pathogenic pegivirus ‘Theiler disease‐associated virus’ (TDAV) and non‐pathogenic equine pegivirus (EPgV) have been identified indicating not all strains of pegivirus are pathogenic (Kapoor et al., 2013; Smith, Chalmers, & Wedel, 1991; Sturgeon, 2017). The recent identifications of PPgV could be associated with pathogenesis, but a clear link of pathogenesis has not been established (Baechlein et al., 2016; Yang et al., 2018). The identification of PPgV in only two of the 10 serum samples suggests PPgV may not be the cause of clinical disease. However, distant PPgV strains are circulating in the US and Germany. Further detection and sequencing of global PPgV strains will elucidate the prevalence and genetic diversity of this newly identified virus in swine.

A novel pestivirus, APPV, was recently discovered and causes CT in piglets (Arruda et al., 2016; de Groof et al., 2016; Postel et al., 2016). Infectivity of piglets during gestation is required to cause CT, and differences in timing of infection and concentration of APPV to piglets in utero are associated with varying severity of CT (Arruda et al., 2016; de Groof et al., 2016). Our APPV positive cases were not associated with CT, highlighting pigs can be asymptomatic carries of APPV. The US strains clustered in different branches within Group I of the phylogenetic tree, indicating genetic diversity within the US. However, the diversity of APPV in greater China since the strains were in different groups of the phylogenetic tree.

In this study, a duplex sqRT‐PCR was developed to determine the occurrence of PPgV and APPV infections in diagnostic samples from US swine farms, following the discovery of these pathogens using NGS. A sqRT‐PCR for PPgV has yet to be described (Baechlein et al., 2016; Yang et al., 2018), whereas a few sqRT‐PCRs are have been described for APPV (Arruda et al., 2016; Gatto et al., 2018; Postel et al., 2016). The duplex sqRT‐PCR had high analytical sensitivity and specificity, which can be used to screen a large number of samples. The PPgV and APPV infections may be associated with clinical diseases in our study, although the cases in this study were asymptomatic. While Koch's postulate has been fulfilled for APPV, Koch's postulate has yet to be established with PPgV. These factors indicate a lack of understanding of the relationship between PPgV with infectivity and pathogenesis. Given these new findings, the pathogenesis, clinical signs and association of PPgV and APPV with other co‐infections should be further investigated in swine.

## CONFLICT OF INTEREST STATEMENT

The authors declare no conflict of interest.
