# Supplementary material for: Semi‐quantitative duplex RT‐PCR reveals the low occurrence of Porcine Pegivirus and Atypical Porcine Pestivirus in diagnostic samples from the United States
Source: Transbound Emerg Dis. 2019 Mar 21;66(3):1420–5. doi: 10.1111/tbed.13154 (PMC6849716; doi:10.1111/tbed.13154)
Supplement: Supplementary file 2 [file TBED-66-1420-s002.pdf]

Appendix Table 1. The primers and probes used in the PPgV and APPV duplex real-time RT-PCR.

Primer or probe name	Primer/probe sequence (5'-3')	Location (bp)	Purpose
Pegi-1F	GCTTGTCGGCAGAGCCC	5,292-5,308	
Pegi-1R	GTTCCTTCCACACCAACCCAT	5,339-5,359	Pegivirus detection
Pegi-Probe-1	5'Cy5-AACTGCAGCCCGCCTTCTGATGATC-3'BHQ1	5,310-5,334	
Pesti-1F	GCAAAGATGCCCTTGATTGTC	5,770-5,790	
Pesti-1R	CTTTTGCCTRCCCACTCG	5,845-5,862	Pestivirus detection
Pesti-Probe-1	5'FAM-AGGGAAGAACAGGGCCAGAGGAAAGG-3'BHQ1	5,818-5,843	
