# Supplementary material for: Semi‐quantitative duplex RT‐PCR reveals the low occurrence of Porcine Pegivirus and Atypical Porcine Pestivirus in diagnostic samples from the United States
Source: Transbound Emerg Dis. 2019 Mar 21;66(3):1420–5. doi: 10.1111/tbed.13154 (PMC6849716; doi:10.1111/tbed.13154)
Supplement: Supplementary file 3 [file TBED-66-1420-s003.docx]

**Supplemental material. Porcine pathogens used for diagnostic specificity.**

Porcine Cytomegalovirus, North American Porcine Reproductive and Respiratory Syndrome, European Porcine Reproductive and Respiratory Syndrome, Pseudorabies Virus, Swine Influenza A virus H1, H2, and H3, Transmissible Gastroenteritis Virus, Porcine Respiratory Coronavirus, Porcine Hemagglutinating Encephalomyelitis Virus, Encephalomyocarditis Virus, Enterovirus, Hog Cholera, Hepatitis E Virus, Porcine Parvovirus Type I, Porcine Adenovirus, Porcine Circovirus Type I and II, Picornavirus, Porcine Rotavirus A, B, and C, Porcine Lymphotropic Gamma Herpes Virus 1, Porcine Lymphotropic Gamma Herpes Virus 2, Porcine Hokovirus, Porcine Epidemic Diarrhea Virus, Beta-hemolytic *Escherichia coli*, Non-beta-hemolytic *E. coli, Pasteurella multocida, Salmonella cholerasuis, S. typhimurium, Bordetella bronchiseptica, Clostridium perfringens* Type A, *C. perfringens* Type C, *Brachyspira hampsonii* Colon A, *B. hampsonii* Colon B, *B. hyodysenteriae, B. pilosicoli , B. murdochii, B. intermedia, B. innocens, Actinobacillus suis, A. rossi, A. minor, A. indolicus, A. equuli, A. pleuropneumoniae, A. pyogenes, Streptococcus suis, Enterococcus durans, Yersinia enterocolitica, Campylobacter coli, C. jejuni, Staphylococcus aureus*, *Haemophilusparasuis*, *Mycoplasma hyorhinis*, *M. hyosynoviae*, and *Erysipelothrix species*.
